# MicroRNA‐497 elevation or LRG1 knockdown promotes osteoblast proliferation and collagen synthesis in osteoporosis via TGF‐β1/Smads signalling pathway

**DOI:** 10.1111/jcmm.15826

**Published:** 2020-09-24

**Authors:** ZhengTao Gu, DengHui Xie, CaiQiang Huang, Rui Ding, RongKai Zhang, QingChu Li, ChuangXin Lin, YiYan Qiu

**Affiliations:** ^1^ Department of Treatment Center For Traumatic Injuries The Third Affiliated Hospital of Southern Medical University Guangdong Provincial Key Laboratory of Bone and Joint Degeneration Diseases Southern Medical University Academy of Orthopedics of Guangdong Province Guangzhou China; ^2^ Division of joint surgery Department of Orthopedics The Third Affiliated Hospital of Southern Medical University Guangdong Provincial Key Laboratory of Bone and Joint Degeneration Diseases Southern Medical University Academy of Orthopedics of Guangdong Province Guangzhou China; ^3^ Division of spine surgery, section Ⅱ Department of Orthopedics The Third Affiliated Hospital of Southern Medical University Guangdong Provincial Key Laboratory of Bone and Joint Degeneration Diseases Southern Medical University Academy of Orthopedics of Guangdong Province Guangzhou China; ^4^ Department of Orthopedic Surgery Shantou Central Hospital Affiliated Shantou Hospital of Sun Yat‐Sen University Shantou P. R. China

**Keywords:** collagen synthesis, leucine‐rich alpha‐2‐glycoprotein‐1, microRNA‐497, osteoblast, osteoporosis, transforming growth factor β1/Smads signalling pathway, viability

## Abstract

MicroRNAs (miRNAs) have been corroborated to engage in the process of cellular activities in osteoporosis. However, few researches have been conducted to expose the integrated role of miR‐497, leucine‐rich alpha‐2‐glycoprotein‐1 (LRG1) and transforming growth factor beta 1 (TGF‐β1)/Smads signalling pathway in osteoporosis. Thereafter, the study is set out to delve into miR‐497/LRG1/TGF‐β1/Smads signalling pathway axis in osteoporosis. Osteoporosis bone tissues and normal bone tissues were collected. Rat osteoporosis models were constructed via ovariectomy. Model rats were injected with restored miR‐497 or depleted LRG1 to explore their roles in osteoporosis. Rat osteoblasts were extracted from osteoporosis rats and transfected with restored miR‐497 or depleted LRG1 for further verification. MiR‐497 and LRG1 expression in femoral head tissues and osteoblasts of osteoporosis rats were detected. TGF‐β1/Smads signalling pathway‐related factors were detected. MiR‐497 was poorly expressed while LRG1 was highly expressed and TGF‐β1/Smads signalling pathway activation was inhibited in osteoporosis. MiR‐497 up‐regulation or LRG1 down‐regulation activated TGF‐β1/Smads signalling pathway, promoted collagen type 1 synthesis and suppressed oxidative stress in femoral head tissues in osteoporosis. MiR‐497 restoration or LRG1 knockdown activated TGF‐β1/Smads signalling pathway, promoted viability and suppressed apoptosis of osteoblasts in osteoporosis. Our study suggests that miR‐497 up‐regulation or LRG1 down‐regulation promotes osteoblast viability and collagen synthesis via activating TGF‐β1/Smads signalling pathway, which may provide a novel reference for osteoporosis treatment.

## 1 INTRODUCTION

Osteoporosis is widely recognized as a bone disease which is manifested with low bone mass and microarchitectural deterioration of bone tissues, resulting in bone fragility and thus posing a great threat on bone fracture.^1^ An enormous number of people of both genders and all races are affected by osteoporosis, and its prevalence will increase in the process of ageing.^2^ There are risk factors contributing to the incidence of osteoporosis, including age, smoking, low weight, alcohol intake, oestrogen status, physical inactivity, low calcium intake and low vitamin D status.^3^ Thus, antiresorptive therapy, tobacco avoidance, adequate combined calcium and vitamin D intake, moderate alcohol intake and weight‐bearing exercise are introduced for patients with osteoporosis.^4^ More importantly, it is essential to recognize the optimal treatment of osteoporosis.

MicroRNAs (miRNAs) are small non‐coding endogenous RNAs regulating gene expression at post‐transcriptional level which interfere with translation of specific target mRNAs and are thought to regulate many cellular processes.^5^ Deregulated miRNA‐mediated mechanisms are found to become the crucial pathological factor in bone deterioration and other bone‐related diseases.^6^ Specifically, circulating miR‐497‐5p is regarded as a promising biomarker for osteoporosis diagnosis and prognosis due to its participation in bone metabolism and bone loss.^7^ A study has elucidated that miR‐497 ~ 195 cluster plays an unique but irreplaceable role in regulating angiogenesis combined with osteoporosis and its function may widen our vision to treat with age‐related osteoporosis.^8^ It has been reported that transforming growth factor β1 (TGF‐β1)/Smads pathway is involved in dexamethasone‐induced osteoporosis progression.^9^, ^10^ Moreover, regulation of TGF‐β1/Smads pathway has been documented to promote bone formation and preclude bone resorption.^11^ Widely, TGF‐β could improve the repair capacity following bone injury through promoting cell division and osteoblast generation, so that the proliferation effect of TGF‐β notably increases the number of osteoblasts.^12^ In addition, a conclusion drawn from a previous investigation points out that leucine‐rich alpha‐2‐glycoprotein‐1 (LRG1) promotes angiogenesis by modulating endothelial TGF‐β signalling pathway.^13^ Wang *et al* have stated that LRG1 induces endothelial cell angiogenesis, mesenchymal stem cell (MSC) migration and bone formation.^14^ Taking the above‐mentioned findings into consideration, the present study was conducted to identify the molecular mechanism of miR‐497/LRG1/TGF‐β1/Smads axis in osteoporosis and their potentials in the progression of osteoporosis.

## 2 MATERIALS AND METHODS

### 2.1 Ethics statement

The study was approved by the Ethics Committee of the Third Affiliated Hospital, Southern Medical University, Academy of Orthopedics of Guangdong Province, and informed written consent was acquired from all patients. All animal experiments went along with the Guide for the Care and Use of Laboratory Animal by International Committees.

### 2.2 Study subjects

Bone mineral density of patients, who were admitted from January 2017 to September 2018, was measured by a dual‐energy X‐ray bone density meter (DEXA, Norland, USA). According to the diagnostic criteria issued by World Health Organization, osteoporosis is diagnosed if the bone mass density T value is greater than −2.5. The inclusive standard was as follows: osteoporosis patients confirmed by DEXA had complete clinical data and imaging data; specimen collection and use were agreed with the informed consent of the patients and the matching control individuals; the patients were free of severe combinations such as malignant tumour, diabetes, hypertension, or heart disease.

Twenty clinically diagnosed osteoporosis patients who had received hip replacement caused by femur neck fracture were collected as an osteoporosis group, of which 17 cases of female and 3 cases of male whereas 20 patients who had received hip replacement caused by bilateral hip dysplasia were collected as a control group, of which 14 cases of female and 6 cases of male. The cancellous bone located at femoral neck was removed by an aseptic rongeur and preserved at −80℃.

### 2.3 Experimental animals

Healthy female Sprague Dawley (SD) rats (n = 70) of specific‐pathogen‐free grade, ageing 3‐4 months and weighting 180‐250 g, were purchased from the Experimental Animal Center of Guangddong (Guangzhou, China). Adaptively raised for 7 d (free food and drinking water, 24 ± 2℃, 12‐h day/night cycle), rats were applied for experiments.

### 2.4 Osteoporosis rat modelling and grouping

Fasted for 8‐12 h, 70 female SD rats were assigned into the control group (n = 8) and osteoporosis model group (n = 62) by random number table. Anaesthetized with 3% pentobarbital sodium at 0.1 mL/100 g, rats were fixed in a prone position, cut off back hair and disinfected on the back skin with iodine wine and alcohol. On both sides of the spine, vertical incisions were made to separate muscles to open the abdominal cavity to perform ovariectomy (ovary, a flesh‐red particles visible on the adipose tissue, pathologically confirmed as ovarian tissues). After that, if there was no active bleeding in abdominal cavity, muscles and skin were stitched and disinfected with 75% alcohol, and 100,000 units of penicillin were injected to prevent infection. Normally raised for 2 months, the rats were administrated. The model rats (n = 56) were divided into 7 groups (n = 8) and injected through their hip joints: model group (normal saline); miR‐497 mimics group (miR‐497 mimics); mimics negative control (NC) group (miR‐497 mimics NC); LRG1‐siRNA group (LRG1 interference plasmid); siRNA‐NC group (LRG1 interference plasmid NC); miR‐497 mimics + LRG1‐overexpression (OE) group (miR‐497 mimics and LRG1 overexpression plasmid); and miR‐497 mimics + OE‐NC group (miR‐497 mimics and LRG1 overexpression plasmidNC ). The above mimics, siRNAs and plasmids were synthesized by GenePharma Ltd. Company (Shanghai, China). Rats were euthanized 2 weeks after injection. Metabolic cages were used to retain urine within 24 hours to detect biochemical indicators. With rats anaesthetized with 3% pentobarbital sodium at 0.1 mL/100 g and fixed on the operating table, the abdominal cavity was opened to obtain abdominal aorta blood (5‐8 mL). The blood samples were centrifuged at 3000 rpm/min to take serum and stored at −70°C for detection of bone formation‐related indicators. Part of femoral head tissues was applied for histological observation, and the other part was preserved in liquid nitrogen for reverse transcription quantitative polymerase chain reaction (RT‐qPCR) and Western blot analysis, etc.

### 2.5 Detection of blood and urine biochemical indices

Urinary calcium and blood calcium were determined by methyl thymol blue colorimetric method, urinary phosphorus and blood phosphorus by reduction by ferrous ammonium sulphate hexahydrate and hydroxyproline (Hyp) by dimethylaminobenzaldehyde method. These kits were provided by Beijing Leagene Biotech. Co., Ltd, (Beijing, China). Alkaline phosphatase (ALP) was detected by aminoantipyrine colorimetric method, and the detection kit was provided by NanJing JianCheng Bioengineering Institute (Nanjing, China).

### 2.6 Detection of bone formation‐related indices

Stood at room temperature for 30 minutes, the blood samples were centrifuged at 3000 r/min to separate serum, in which osteocalcin (OC), bone alkaline phosphatase (BALP), aminoterminal (PINP) and carboxyterminal (PICP) contents were determined by enzyme‐linked immunosorbent assay (ELISA) kits (Shanghai Enzyme‐linked Biotechnology Co., Ltd., Shanghai, China). Optical density (OD) values were measured at 450 nm (within 10 minutes after termination) on a microplate reader (infinite M200, Tecan, Austria). A standard curve was drawn using a computer CurveExpert 1.3 analysis software to calculate the sample content.

### 2.7 Haematoxylin‐eosin (HE) staining

The femoral head tissues were fixed in 10% formalin solution for 24 h and decalcified with 10% ethylene diamine tetraacetic acid (pH = 7.2). Then, the samples were sequentially dehydrated in ethanol of different concentrations, permeabilized by xylene, embedded in paraffin and sectioned into 5 μm. The paraffin sections were placed in xylene I and xylene II, treated with gradient ethanol and stained with haematoxylin solution. Next, the sections were differentiated with 1% hydrochloric acid alcohol solution, treated with 1% ammonia water and counterstained with 1% eosin solution. Followed by that, the sections were dehydrated, permeabilized, sealed with neutral resin and analysed by IPP6.0 software (Media Cybernetics, USA).

### 2.8 Detection of oxidative stress‐related indices

The preserved femoral head tissues in liquid nitrogen were diluted with normal saline, homogenized and centrifuged at 3000 r/min to obtain the supernatant. Superoxide dismutase (SOD) activity was determined by xanthine oxidase method, malondialdehyde (MDA) content by thiobarbituric acid method and glutathione peroxidase (GSH‐Px) activity by colorimetric method. The SOD, MDA and GSH‐Px kits were purchased from Nanjing Jiancheng Bioengineering Institute.

### 2.9 Osteoblast isolation, identification and treatment

Isolation of osteoblasts: Under aseptic conditions, cancellous bone was rinsed 3 times with sterile phosphate‐buffered saline (PBS), shred into approximately 1 mm^3^ bone particles and rinsed with PBS until the bone particles turned into white. Bone particles were detached with 0.1% collagenase at 1:10 at 37°C water bath for 20 minutes and detached with collagenase again for about 1 h. The suspension obtained by detachment was centrifuged at 1200 r/min, rinsed 3 times with PBS and added with a culture solution containing 15% newborn bovine serum, penicillin (100 U/mL) and streptomycin (100 U/mL) to make a suspension. Incubated at 37°C, 5% CO_2_ with saturated humidity, osteoblasts were fully adhered, with the medium renewed every 3 days.

Identification of osteoblasts: (1) Morphological observation: The growth and morphological changes of primary and sub‐cultured osteoblasts were observed under an inverted phase contrast microscope and photographed in randomly selected fields of views. (2) ALP staining: Osteoblasts of passage 2 were cultured for 7 days and ALP staining was carried out referring to the instructions of the ALP staining kit (NanJing JianCheng Bioengineering Institute). Osteoblasts were deprived of culture medium and fixed with fixation solution for 3 minutes. Followed by that, the osteoblasts were covered by the freshly prepared substrate application solution and with a hydrophobic membrane. Incubated in a wet box at 37°C in the dark, the osteoblasts were counterstained with haematoxylin and observed by a microscope.

Osteoblasts from osteoporosis rats were distributed into 7 groups: model group (osteoporosis rat osteoblasts without any treatment); miR‐497 mimics group (osteoblasts transfected with miR‐497 mimics), mimics NC group (osteoblasts transfected with miR‐497 mimics NC); LRG1‐siRNA group (osteoblasts transfected with LRG1 interference vector); siRNA‐NC group (osteoblasts transfected with LRG1 interference vector NC); miR‐497 mimics + LRG1‐OE group (osteoblasts transfected with miR‐497 mimics and LRG1 overexpression vector); and miR‐497 mimics + OE‐NC group (osteoblasts transfected with miR‐497 mimics and LRG1 overexpression vector NC ). A normal group was set with normal SD rat osteoblasts without any treatments. The above mimics, siRNAs and vectors were synthesized by GenePharma.

### 2.10 3‐(4,5‐Dimethylthiazol‐2‐yl)‐2,5‐diphenyltetrazolium bromide (MTT) assay

Detached with trypsin and seeded in 96‐well plates, osteoblasts of concentration of 4 × 10^4^ cells/well (3 replicates for each group) were deprived of culture medium on the 0, 12th, 24th, 36th and 48th h, respectively, and supplemented with 200 μL 5 g/L MTT solution (Sigma, St. Louis, MO, USA). Next, the osteoblasts were incubated with 150 μL dimethyl sulphoxide solution without light exposure. OD values were measured at 490 nm on a microplate reader (infinite M200, Tecan).

### 2.11 Flow cytometry

Propidium iodide (PI) single staining for cell cycle distribution: After 48 h of transfection, the cells were centrifuged and triturated into cell suspension. Then, the cells were fixed with 70% ice ethanol, centrifuged and triturated into cell suspension with 4°C PBS. After that, the cells were successively incubated with 150 μL RNase A (Sigma) and 150 μL PI (Sigma). Cell cycle distribution was tested by a flow cytometer (Thermo Fisher Scientific, Waltham, MA, USA).

Annexin V‐APC/PI double staining for cell apoptosis: Binding buffer was diluted 4 times with deionized water (4 mL binding buffer + 12 mL deionized water). Immersed twice in pre‐cooled PBS and resuspended in 250 μL binding buffer to 1 × 10^6^ cells/mL, the cells (100 μL) were incubated with 5 μL Annexin V‐APC (BD Biosciences, New Jersey, USA) and 5 μL PI solution (BD Biosciences). Followed by that, the cells were supplemented with 400 μL PBS and detected on a flow cytometer with the results automatically analysed by a computer.

### 2.12 RT‐qPCR

Total RNA in tissues and cells were extracted by TRIzol extraction kit (Invitrogen, California, USA). Primers were synthesized by Sangon Biotech Co., Ltd. (Shanghai, China) (Table 1). PrimeScript RT test kit was applied to finish reverse transcription of RNA into complementary DNA (cDNA). With cDNA as a template, fluorescence quantitative PCR was processed by ExiLENT SYBR Green Master Mix (Exiqon, Beverly, MA, USA). U6 was regarded as a loading control for miRNA while β‐actin for other genes. 2^−△△Ct^ method was used for quantitative analysis.^15^

**TABLE 1:** Primer sequence

Gene	Primer sequence (5’‐ 3’)
miR‐497	Forward: AGTCCAGTTTTCCCAGGAATCCCT
	Reverse: ACCAGCAGCACACTGTGGTTTGT
miR‐195	Forward: CGTAGCAGCACAGAAAT
	Reverse: GTGCAGGGTCCGAGGT
LRG1	Forward: CCTTACCCATGCCCAAGGTG
	Reverse: CTGGGAGAACCCAGAGTTCAAGAG
U6	Forward: CTCGCTTCGGCAGCACA
	Reverse: AACGCTTCACGAATTTGCGT
β‐actin	Forward: TTCTTTGCAGCTCCTTCG
	Reverse: TCTCCATGCGCCCAGT

Note

miR‐497, microRNA‐497; miR‐195, microRNA‐195; LRG1, leucine‐rich alpha‐2‐glycoprotein‐1.

### 2.13 Western blot analysis

Total proteins were extracted from bone tissues and cells, followed by protein concentration determination and deionized water adjustment. The 10% sodium dodecyl sulphate separation gel and concentrated gel were prepared. Mixed with the loading buffer, the protein samples were boiled at 100°C which was followed by ice bath and centrifugation. Then, the protein samples were processed with electrophoresis separation and electroblotting onto a nitrocellulose membrane. Next, the membrane was blocked with 5% skim milk powder overnight and probed with the primary antibodies LRG1 (1:1000, Proteintech, Chicago, IL, USA), collagen type 1 (Col‐1, 1:1000), phosphorylated (p)‐Smad2/3 (1:1000, both from Abcam, Cambridge, UK), TGF‐β1 (1:1000, R&D Systems, Minneapolis, MN, USA), Smad3 (1:1000, Cell Signaling Technology, Beverly, MA, USA), Smad4 (1:1000), Smad7 (1:1000), Bcl‐2 (1:1000), Bax (1:1000, Abcam) Cyclin D1 (1:1000) and CDK4 (1:1000, all from Santa Cruz Biotechnology, Santa Cruz, CA, USA). Then, the membrane was reprobed with secondary antibody horseradish peroxidase‐labelled immunoglobulin G (1:1000, Boster Biological Technology Co., Ltd., Wuhan, China). Subsequently, the membrane was immersed in an enhanced chemiluminescence reaction solution (Pierce, Rockford, IL, USA), followed by development and fixation in the dark. With β‐actin (1:1000, Santa Cruz Biotechnology) as an internal control, the protein imprinted image was analysed by ImageJ2x software.

### 2.14 Dual‐luciferase reporter gene assay

An online website (https://cm.jefferson.edu/rna22/Precomputed/) was in application to prediction of the possible binding sites of miR‐497 and LRG1. The 3’ untranslated region (UTR) fragment of LRG1 was cloned into a pmirGLO plasmid (Promega, Wisconsin, USA) to construct a pmirGLO‐LRG1‐wild‐type (WT) plasmid. A site‐directed mutation kit (Takara, Tokyo, Japan) was indicated to mutate the miR‐497 binding site on the LRG1 3’UTR to construct a pmirGLO‐LRG1‐mutant (MUT) plasmid. The plasmids and miRNA were transfected into rat osteoblasts with reference to the instructions of Lipofectamine 2000. The pmirGLO‐LRG1‐WT plasmid or pmirGLO‐LRG1‐MUT and miR‐497 mimic or mimic NC were cotransfected into cells. Cells were collected 48 h later, and the dual‐luciferase activity detection kit (Promega) was implied to detect luciferase activity.

### 2.15 Statistical analysis

Data analysis was conducted by SPSS21.0 software (IBM, New York, NY, USA). Data were expressed as mean ± standard deviation. The data were firstly evaluated by Kolmogorov‐Smirnov and Levene's methods. The data that were normally distributed and in heterogeneity of variance were analysed with *t* test (two groups), one‐way analysis of variance (ANOVA; multiple groups) and Tukey's post hoc test (pairwise comparison). Otherwise, the data were assessed by Mann‐Whitney U test. Correlation analysis was performed by Pearson's test. All test were two‐sided, and the difference was of statistical significance at *P* < 0.05.

## 3 RESULTS

### 3.1 MiR‐497 expression reduces and LRG1 expression increases in femoral head tissues of patients with osteoporosis

**FIGURE 1:**
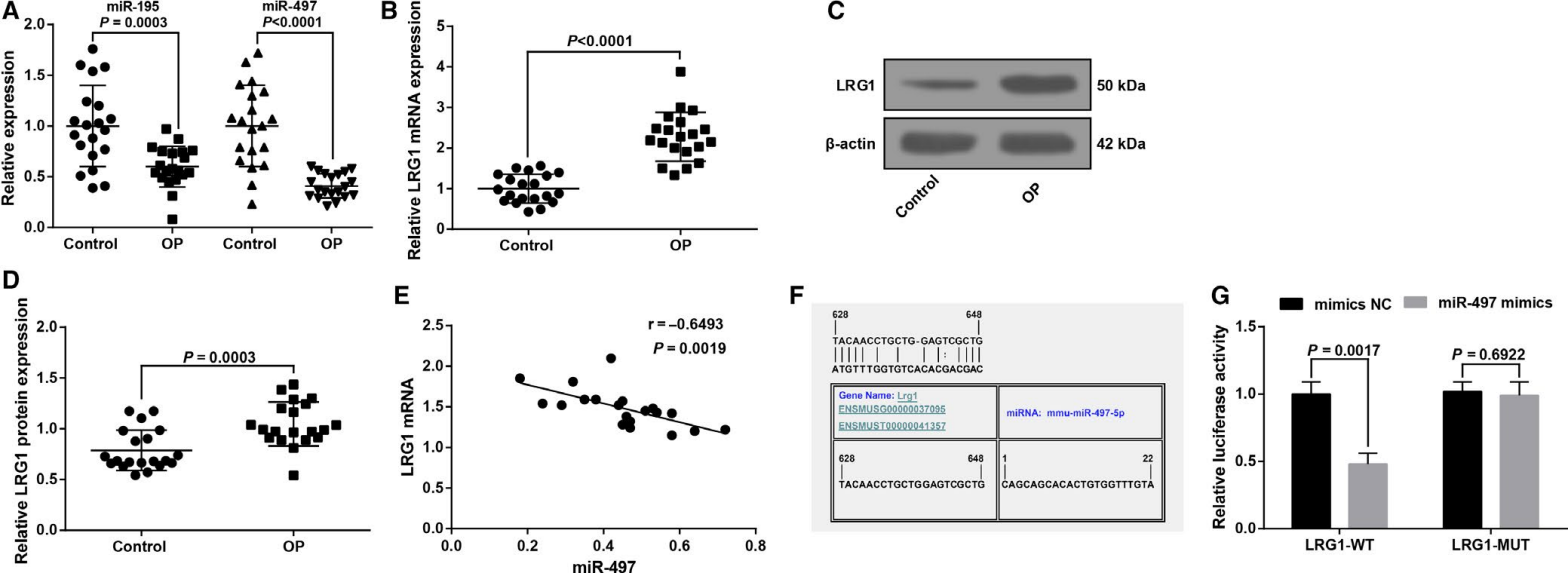
MiR‐497 expression reduces and LRG1 expression increases in femoral head tissues of patients with osteoporosis. A, MiR‐497 expression in femoral head tissues in control and osteoporosis; B, LRG1 mRNA expression in femoral head tissues in control and osteoporosis; C, LRG1 protein bands in femoral head tissues in control and osteoporosis; D, LRG1 protein expression in femoral head tissues in control and osteoporosis; E, Pearson's analysis of the correlation between miR‐497 and LRG1 in femoral head tissues in osteoporosis patients; F, schematic diagram of the miR‐497 binding site in the LRG1 based on RNA22 software; G, luciferase activity verification; in figure A‐E, control group (n = 20), osteoporosis group (n = 20); in Figure G, N = 3. The data were expressed as the mean ± standard deviation. Comparisons between two groups were analysed by unpaired t test; In Figure E, Pearson correlation analysis was performed

RT‐qPCR results (Figure 1A) showed that miR‐497 and miR‐195 expression in femoral head tissues of the osteoporosis patients were reduced (all *P* < 0.05) in contrast to controls. As miR‐497 showed a more obvious difference, miR‐497 was selected for the studied miRNA.

RT‐qPCR and Western blot analysis demonstrated that LRG1 expression was increased in femoral head tissues of the osteoporosis patients by comparison with controls (Figure 1B‐D).

A negative relation was observed between miR‐497 and LRG1 (r = −0.6493, *P* = 0.0019) in femoral head tissues of osteoporosis patients by Pearson's correlation analysis (Figure 1E).

RNA22 tool (https://cm.jefferson.edu/rna22/Precomputed/) ^16^ predicted that miR‐497 could bind to LRG1 (Figure 1F). In order to confirm that LRG1 was a direct target gene of miR‐497, dual‐luciferase reporter gene assay was implemented with the results suggesting that versus cells cotransfected with mimics NC + LRG1‐WT, the luciferase activity of cells cotransfected with miR‐497 mimics + LRG1‐WT was impaired (*P* < 0.05). No discrepancy was identified between cells cotransfected mimics NC + LRG1‐MUT and miR‐497 mimics + LRG1‐MUT (*P* > .05), implying that LRG1 was a target gene regulated by miR‐497 (Figure 1G).

### 3.2 Up‐regulation of miR‐497 or down‐regulation of LRG1 activates TGF‐β1/Smads signalling pathway in rats with osteoporosis

**FIGURE 2:**
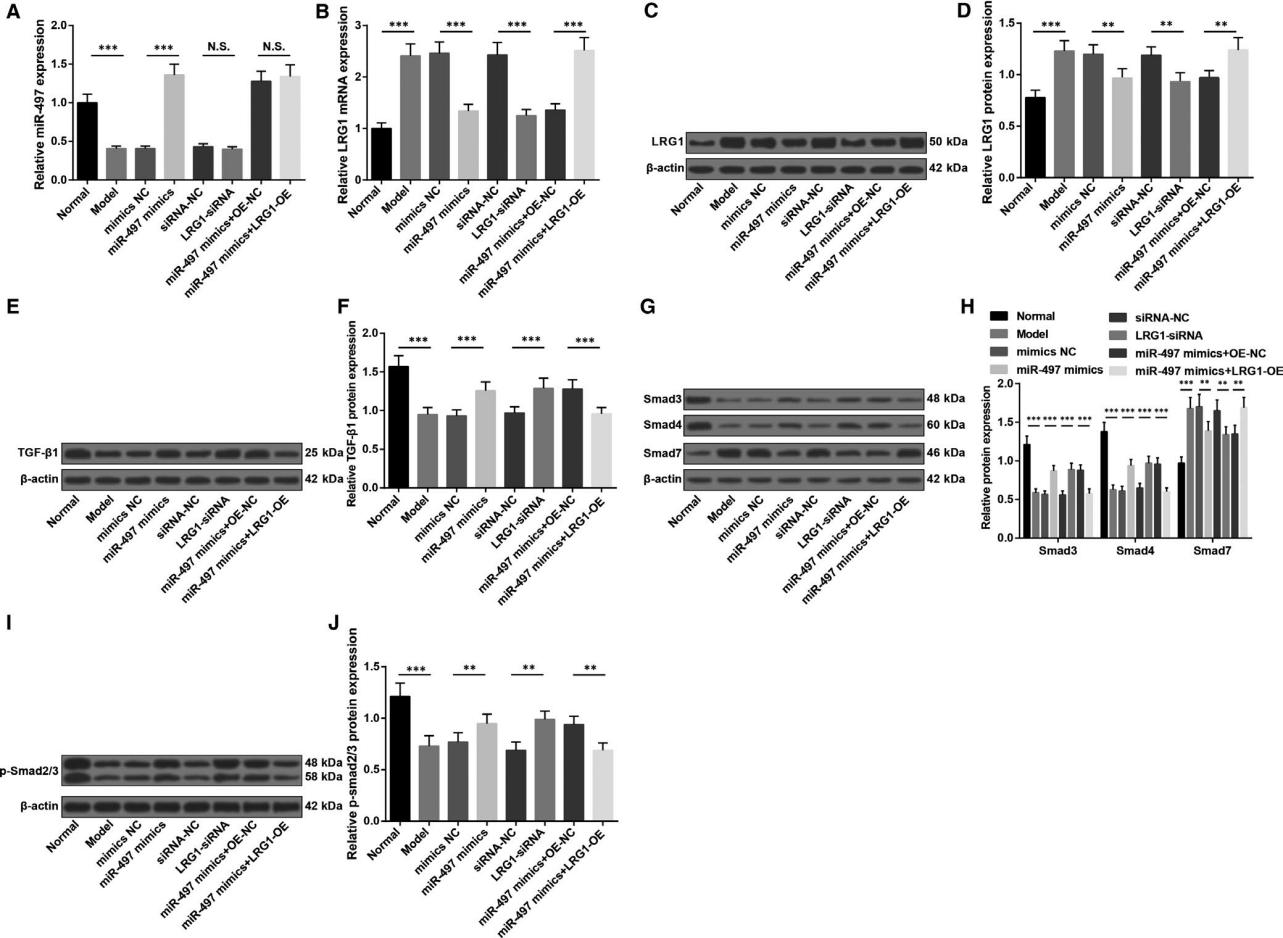
Up‐regulation of miR‐497 or down‐regulation of LRG1 activates TGF‐β1/Smads signalling pathway in rats with osteoporosis. A, MiR‐497 expression in femoral head tissues of rats; B, LRG1 mRNA expression in femoral head tissues of rats; C, LRG1 protein bands in femoral head tissues of rats; D, LRG1 protein expression in femoral head tissues of rats; E, TGF‐β1 protein bands in femoral head tissues of rats; F, TGF‐β1 protein expression in femoral head tissues of rats; G, Smad3, Smad4 and Smad7 protein bands in femoral head tissues of rats; H, Smad3, Smad4 and Smad7 protein expression in femoral head tissues of rats; I, p‐Smad2/3 protein bands in femoral head tissues of rats; J, p‐Smad2/3 protein expression in femoral head tissues of rats; * represented *P* < 0.05; **represented *P* < 0.01; *** represented *P* < 0.001; n = 8. The data were expressed as the mean ± standard deviation. Comparisons among multiple groups were analysed using ANOVA, followed by Tukey's post hoc test for pairwise comparisons

RT‐qPCR and Western blot analysis (Figure 2A‐D) demonstrated that in bone tissues, miR‐497 expression down‐regulated while LRG1 expression elevated in the osteoporosis rats in comparison with the normal SD rats (all *P* < 0.05), indicating the involvements of miR‐497 and LRG1 in osteoporosis. In osteoporosis rats, up‐regulation of miR‐497 or down‐regulation of LRG1 lowered LRG1 expression, which further suggested the participation of miR‐497 in osteoporosis by down‐regulating LRG1.

To further explore the effects of up‐regulating miR‐497 or down‐regulating LRG1 on TGF‐β1/Smads signalling pathway, Western blot analysis was adopted to detect TGF‐β1, Smad3, p‐Smad2/3, Smad4 and Smad7 protein expression in rat bone tissues. The results elucidated that (Figure 2E‐J) in relation to normal SD rats, TGF‐β1, Smad3, Smad4 and p‐Smad2/3 expression reduced while Smad7 expression increased in osteoporosis rats (all *P* < 0.05). Up‐regulating miR‐497 or down‐regulating LRG1 could reduce Smad7 while increase TGF‐β1, Smad3, Smad4 and p‐Smad2/3 expression (all *P* < 0.05). Up‐regulating LRG1 reversed the impacts of up‐regulated miR‐497 on TGF‐β1/Smads signalling pathway‐related proteins.

### 3.3 Up‐regulation of miR‐497 or down‐regulation of LRG1 increases blood calcium and blood phosphorus levels, reduces urinary calcium, urinary phosphorus, Hyp and ALP levels, and lowers OC, BALP, PINP and PICP contents in rats with osteoporosis

**FIGURE 3:**
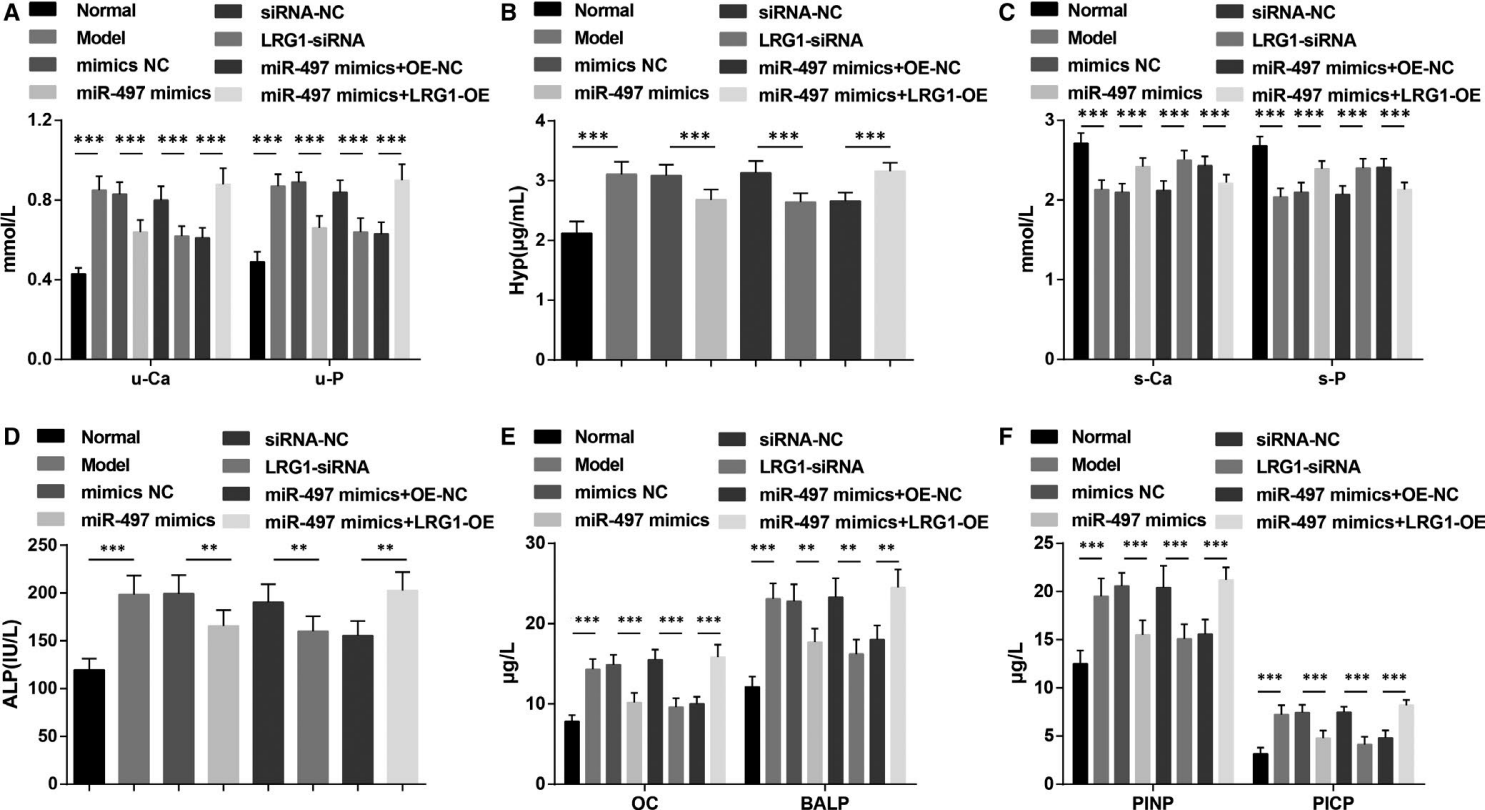
Up‐regulation of miR‐497 or down‐regulation of LRG1 increases blood calcium and blood phosphorus levels, reduces urinary calcium, urinary phosphorus, Hyp and ALP levels and lowers OC, BALP, PINP and PICP contents in rats with osteoporosis. A, Urinary calcium and phosphorus levels of rats; B, urinary Hyp of rats; C, blood calcium and phosphorus levels of rats; D, blood ALP content of rats; E, OC and BALP contents in blood of rats; F, PINP and PICP contents in blood of rats; * represented *P* < 0.05; **represented *P* < 0.01; *** represented *P* < 0.001; n = 8. The data were expressed as the mean ± standard deviation. Comparisons among multiple groups were analysed using ANOVA, followed by Tukey's post hoc test for pairwise comparisons

Detection of haematuria biochemical indices revealed (Figure 3A‐D) that versus the normal and miR‐497 mimics + OE‐NC groups, the increments in urinary calcium, urinary phosphorus, Hyp and ALP levels and the reductions in blood calcium and blood phosphorus levels were manifested in the model and miR‐497 mimics + LRG1‐OE groups (all *P* < 0.05), which were on the opposite way of the situation in the miR‐497 mimics and LRG1‐siRNA groups by comparison with their NC groups (all *P* < 0.05).

ELISA results (Figure 3E and F) showed that the protein levels of OC, BALP, PINP and PICP in rat blood increased in the model and miR‐497 mimics + LRG1‐OE groups in contrast to the normal and miR‐497 mimics + OE‐NC groups (all *P* < 0.05). However, rats injected with miR‐497 mimics and LRG1‐siRNA were featured by declined OC, BALP, PINP and PICP expression (all *P* < 0.05).

### 3.4 Up‐regulation of miR‐497 or down‐regulation of LRG1 attenuates pathological femoral tissue damage, raises Col‐1 expression and inhibits oxidative stress in femoral head tissues of rats with osteoporosis

**FIGURE 4:**
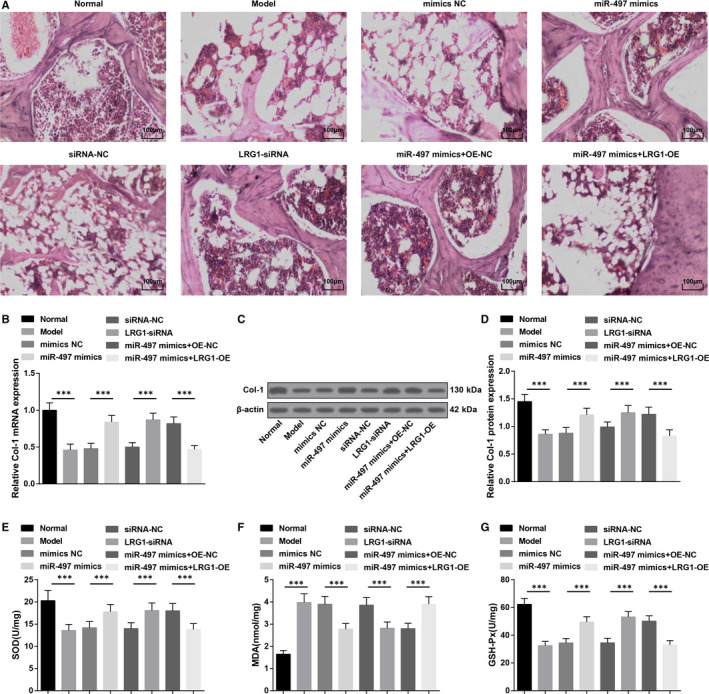
Up‐regulation of miR‐497 or down‐regulation of LRG1 attenuates pathological femoral tissue damage, raises Col‐1 expression and inhibits oxidative stress in femoral head tissues of rats with osteoporosis. A, HE staining of femoral head tissues of rats; B, Col‐1 mRNA expression in femoral head tissues of rats; C, Col‐1 protein bands in femoral head tissues of rats; D, Col‐1 protein expression in femoral head tissues of rats; E, SOD activities in rat femoral head tissues; F, MDA contents in rat femoral head tissues; G, GSH‐Px activities in rat femoral head tissues; * represented *P* < 0.05; **represented *P* < 0.01; *** represented *P* < 0.001; n = 8. The data were expressed as the mean ± standard deviation. Comparisons among multiple groups were analysed using ANOVA, followed by Tukey's post hoc test for pairwise comparisons

HE staining depicted (Figure 4A) that the femoral skull trabeculae in the normal group were thick, neatly arranged, relatively complete in morphology and structure with intact bone marrow cavity. In the model group, the trabeculae were broken, cluttered and the morphology was incomplete, and the bone marrow cavity was hollow. The phenomenon demonstrated in the mimics NC, siRNA‐NC, miR‐497 mimics + LRG1‐OE groups was similar to that in the model group. The miR‐497 mimics, LRG1‐siRNA and miR‐497 mimics + OE‐NC groups were pictured with reduced bone marrow cavity and elevated trabeculae with intact structure and neat arrangement by comparison with the model group.

RT‐qPCR and Western blot analysis manifested that (Figure 4B‐D) lowly expressed Col‐1 in femoral head tissues in osteoporosis rats was increased by transfection with miR‐497 mimics or LRG1‐siRNA (all *P* < 0.05). LRG1‐OE could down‐regulate miR‐497 mimics‐caused upregulated Col‐1 expression (*P* < 0.05).

SOD and GSH‐Px activities and MDA content in rat femoral head tissues presented that (Figure 4E‐G) in comparison with the normal and miR‐497 mimics + OE‐NC groups, SOD and GSH‐Px activities impaired and MDA content elevated in the model and miR‐497 mimics + LRG1‐OE groups (all *P* < 0.05). MiR‐497 mimics or LRG1‐siRNA could lead to the enhancements in SOD and GSH‐Px activities and a decline in MDA content (all *P* < 0.05).

### 3.5 MiR‐497 is down‐regulated while LRG1 is up‐regulated in osteoblasts of rats with osteoporosis, and miR‐497 elevation or LRG1 depletion activates TGF‐β1/Smads signalling pathway

**FIGURE 5:**
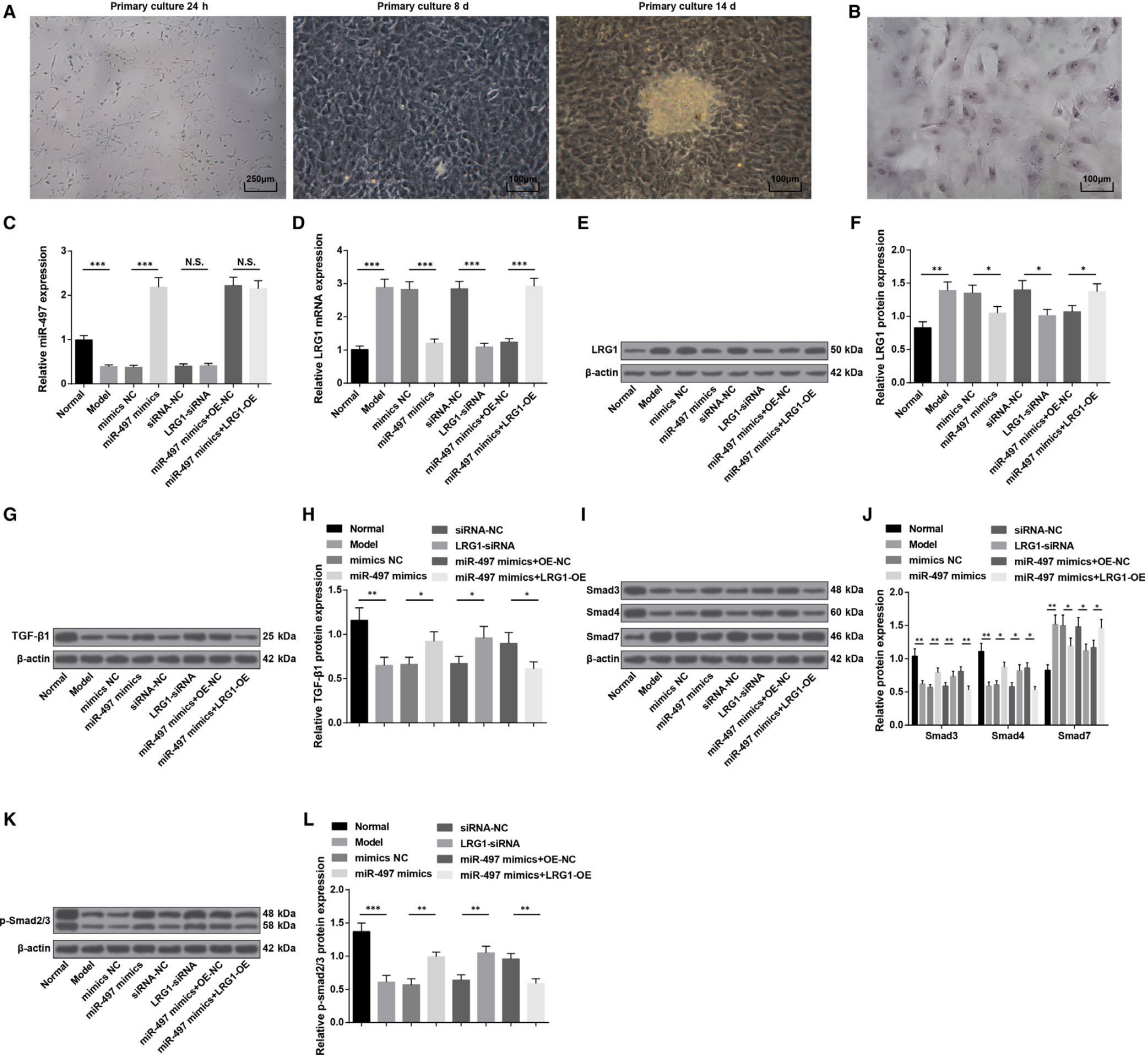
MiR‐497 is down‐regulated while LRG1 is up‐regulated in osteoblasts of rats with osteoporosis, and miR‐497 elevation or LRG1 depletion activates TGF‐β1/Smads signalling pathway. A, Osteoblast morphology at different time‐points; B, ALP staining of osteoblasts; C, MiR‐497 expression in rat osteoblasts; D, LRG1 mRNA expression in rat osteoblasts; E, LRG1 protein bands in rat osteoblasts; F, LRG1 protein expression in rat osteoblasts; G, TGF‐β1 protein band in rat osteoblasts; H, TGF‐β1 protein expression in rat osteoblasts; I, Smad3, Smad4 and Smad7 protein bands in rat osteoblasts; J, Smad3, Smad4 and Smad7 protein expression in rat osteoblasts; K, p‐Smad2/3 protein bands in rat osteoblasts; L, p‐Smad2/3 protein expression in rat osteoblasts; * represented *P* < 0.05; **represented *P* < 0.01; *** represented *P* < 0.001; N = 3. The data were expressed as the mean ± standard deviation. Comparisons among multiple groups were analysed using ANOVA, followed by Tukey's post hoc test for pairwise comparisons

ALP staining (Figure 5A and B): After ALP staining, it could be seen that the cell membrane and intracytoplasmic particles were stained red‐brown, that was, the cell staining was positive, so the successful culture of osteoblasts was identified.

RT‐qPCR and Western blot analysis (Figure 5C‐F) highlighted that reduced miR‐497 and increased LRG1 were characterized in the model group versus the normal group (both *P* < 0.05). MiR‐497 mimics elevated miR‐497 expression and degraded LRG1 expression (both *P* < 0.05). LRG1‐siRNA has no effects on miR‐497 expression (*P* >.05) but reduced LRG1 expression in osteoblasts (*P* < 0.05). Versus the miR‐497 mimics + OE‐NC group, no divergence was identified in miR‐497 expression in the miR‐497 mimics + LRG1‐OE group (*P* > .05), and LRG1 expression increased (*P* < 0.05).

Furthermore, Western blot analysis revealed that (Figure 5G‐L) TGF‐β1, Smad3, Smad4 and p‐Smad2/3 expression declined while Smad7 expression elevated in osteoblasts of osteoporosis rats (all *P* < 0.05). Restoring miR‐497 or silencing LRG1 could reduce Smad7 expression and increase TGF‐β1, Smad3, Smad4 and p‐Smad2/3 expression (all *P* < 0.05). LRG1 up‐regulation could reverse the effects of up‐regulated miR‐497 on TGF‐β1/Smads signalling pathway‐related proteins.

### 3.6 Up‐regulation of miR‐497 or down‐regulation of LRG1 inhibits osteoblast apoptosis and promotes Col‐1 synthesis of rats with osteoporosis

**FIGURE 6:**
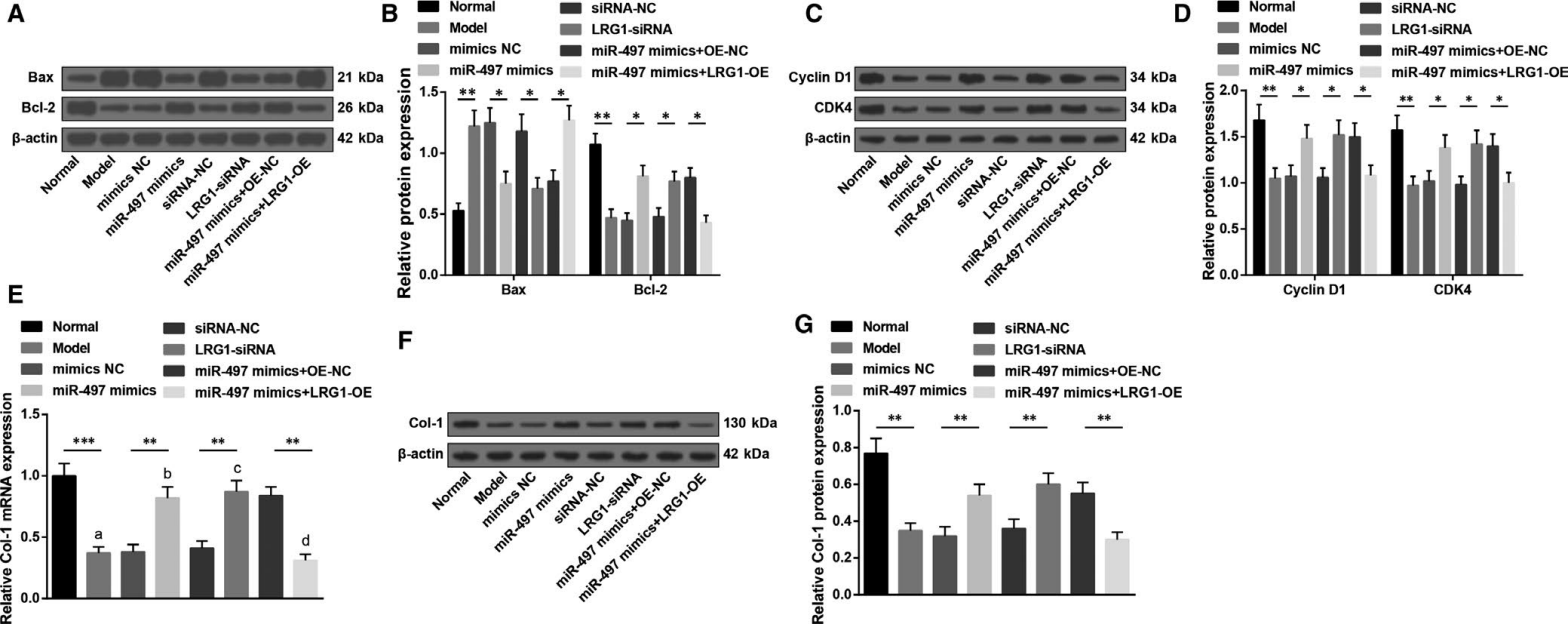
Up‐regulation of miR‐497 or down‐regulation of LRG1 inhibits osteoblast apoptosis and promotes Col‐1 synthesis of rats with osteoporosis. A, Protein bands of Bax and Bcl‐2 in rat osteoblasts; B, Bax and Bcl‐2 protein expression in rat osteoblasts; C, protein bands of Cyclin D1 and CDK4 in rat osteoblasts; D, cyclin D1 and CDK4 protein expression in rat osteoblasts; E, Col‐1 mRNA expression in rat osteoblasts; F,Col‐1 protein bands in rat osteoblasts; G, Col‐1 protein expression in rat osteoblasts; * represented *P* < 0.05; **represented *P* < 0.01; *** represented *P* < 0.001; N = 3. The data were expressed as the mean ± standard deviation. Comparisons among multiple groups were analysed using ANOVA, followed by Tukey's post hoc test for pairwise comparisons

Detected by RT‐qPCR and Western blot analysis (Figure 6A‐G), in osteoblasts, Bcl‐2, Cyclin D1, CDK4 and Col‐1 expression reduced while Bax expression elevated in the model and miR‐497 mimics + LRG1‐OE groups versus the normal and miR‐497 mimics + OE‐NC groups (all *P* < 0.05). However, miR‐497 mimics or LRG1‐siRNA elevated Bcl‐2, Cyclin D1, CDK4 and Col‐1 expression while declined Bax expression versus their corresponding NC (all *P* < 0.05).

### 3.7 Up‐regulation of miR‐497 or down‐regulation of LRG1 promotes viability and suppresses apoptosis in osteoblasts of rats with osteoporosis

**FIGURE 7:**
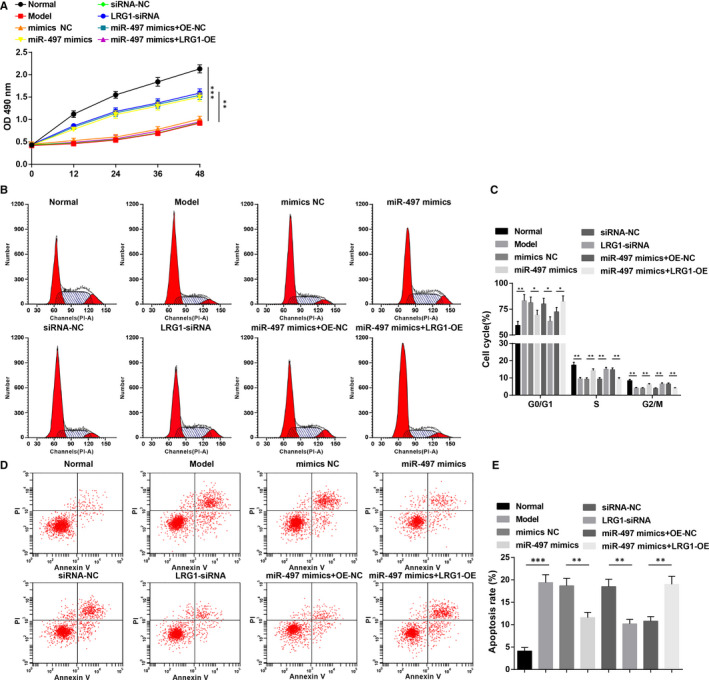
Up‐regulation of miR‐497 or down‐regulation of LRG1 promotes viability and suppresses apoptosis in osteoblasts of rats with osteoporosis. A, Osteoblast viability detected by MTT assay; B, cell cycle distribution of osteoblasts; C, proportion of osteoblast cell cycle; D, osteoblast apoptosis; E, apoptosis rate of osteoblasts; * represented *P* < 0.05; **represented *P* < 0.01; *** represented *P* < 0.001; N = 3. The data were expressed as the mean ± standard deviation. Comparisons among multiple groups were analysed using ANOVA, followed by Tukey's post hoc test for pairwise comparisons

Tested by MTT assay, PI single staining and Annexin V‐APC/PI double staining, it was investigated that (Figure 7A‐E) in osteoblasts, viability was impaired and apoptosis rate was boosted (more G0/G1 and less S and G2/M phases cells) in osteoporosis rats (all *P* < 0.05). Transfection with miR‐497 mimics or LRG1‐siRNA, osteoblasts were demonstrated with enhanced viability as well as reduced apoptosis rate (reduced G0/G1 and increased S and G2/M phases cells) (all *P* < 0.05). It was suggested that overexpressing LRG1 mitigated miR‐497 up‐regulation‐induced enhancement in osteoblast viability and impairment in osteoblast apoptosis.

## 4 DISCUSSION

Osteoporosis is a systemic skeletal disease with variable risk factors and a considerably common chronic condition of bones.^17^ A comparative study has explored the role of miRNAs in the bone development and their therapeutic potentials in osteoporosis,^18^ whereas the unique role of miR‐497 still remains to be scrutinized. Given that, this study is initiated and elucidates that up‐regulation of miR‐497 or down‐regulation of LGR1 promoted osteoporosis osteoblast viability, enhanced collagen synthesis and inhibited osteoblast apoptosis, which may be connected with the activation of TGF‐β1/Smads signalling pathway.

One of the most significant findings demonstrated that miR‐497 expression reduced and LRG1 increased in osteoporosis. It is previously documented that miR‐497‐5p expression was declined in bone tissues of ageing and ovariectomised mice.^7^ Some studies are in accordance with the present study that miR‐497 expression was reduced in breast cancer and in multiple myeloma samples and cell lines ^19^, ^20^ while LRG1 expression was up‐regulated in hepatocellular carcinoma and in ovarian cancer.^21^, ^22^ Riveting on our findings, up‐regulation of miR‐497 or down‐regulation of LRG1 could activate TGF‐β signalling pathway as accompanied by increased TGF‐β1, Smad3, Smad4 and p‐Smad2/3 protein expression and reduced Smad7 protein expression in osteoporosis. A previous study states that the reduced expression of LRG1 is connected to the increased TGF‐β1 activity during the cardiac remodelling process in response to injury.^23^ Moreover, LRG1 deletion can lead to excessive activation of TGF‐β signalling pathway, leading to increased expression of TGF‐β1 and p‐Smad2/3.^24^ A strong relationship between miR‐497 and Smad7 has been recorded in the literature that the expression of miR‐497 was inversely correlated with the Smad7 expression in breast cancer tissues and oral squamous cell carcinoma.^25^, ^26^

In addition, our study also suggested that up‐regulation of miR‐497 or down‐regulation of LRG1 lowered OC, BALP, PINP and PICP contents, and promoted Col‐1 synthesis in osteoporosis. OC is synthesized in the skeleton by osteoblasts with high sensitivity to bone formation, functioning as a prominent marker of bone turnover and diagnosis and follow‐up of high turnover osteoporosis.^27^ BALP, PINP and PICP are all available biochemical markers of bone formation among which BALP is a osteoblast surface marker and serum PICP concentration is the index of metastatic bone disease with potential guidelines to treatments.^28^ PINP is a sensitive indicator of the synthesis of Col‐1. Besides, it is an outstanding indicator of disease activity in bone diseases which is influenced by bone metabolism changes.^29^ As we all know, LRG expression is connected with bone destruction, granulomatous tissue formation and cartilage degeneration.^30^ Furthermore, a study has revealed that LRG1 is donated to angiogenesis‐coupled de novo bone formation.^14^ Besides that, this study has also concluded that miR‐497 restoration or LRG1 knockdown impaired oxidative stress, enhanced cell viability and inhibited apoptosis of osteoblasts in osteoporosis. A previous study infers that aged female rats in osteoporosis exhibited lower serum ALP, a osteoblast differentiation marker gene, and OC level, accompanied with lower serum levels of antioxidants including GSH‐Px activity.^31^ Another study has revealed that female with osteoporosis presented with lower GSH‐Px and SOD enzyme activity and higher level of MDA.^32^ Concerning to the study performed previously, miR‐497 with up‐regulated expression is documented to possess target genes to promote proliferative activity, which is indicative of the promoting function of up‐regulated miR‐497 in proliferative activity.^33^ In addition, another research outcomes state that overexpressed miR‐497 can promote the proliferation capability of cardiomyocyte and inhibit apoptosis of cardiomyocyte in myocardial ischaemia‐reperfusion injury.^34^ Moreover, it is reported that miR‐497 restoration impaired lung cancer cell viability and colony‐forming ability by activating TGF‐β signalling.^35^ Commonly discussed in cancers, LRG1 reduction is surveyed to inhibit cell viability and promote apoptosis of leukaemia cells.^36^ Mechanistically, silencing of LRG1 inhibits oesophageal squamous cell carcinoma cell proliferation and facilitates apoptosis.^37^

Moreover, our study has revealed that miR‐497/LRG1 axis mediated TGF‐β1/Smads pathway to affect osteoporosis. Currently, the activation of TGF‐β/Smad signalling pathway prevented osteoporosis via enhanced bone formation and disrupted bone resorption.^9^ Besides, activating TGF‐β1/Smads pathway was also functional for attenuating glucocorticoid‐induced osteoporosis.^11^ Functionally, the activated TGF‐β1/Smads signalling pathway induced by miR‐202‐3p up‐regulation was indicated to ameliorate oxidative stress and cell apoptosis in myocardial ischaemic‐reperfusion injury.^38^ In papillary thyroid carcinoma, promoting the activation of TGF‐β1/Smads signalling pathway enhanced malignant cell apoptosis and impaired cell proliferation.^39^ Anyway, the activated TGF‐β1/Smads signalling pathway has shown its conducive effects on diseases.

**FIGURE 8:**
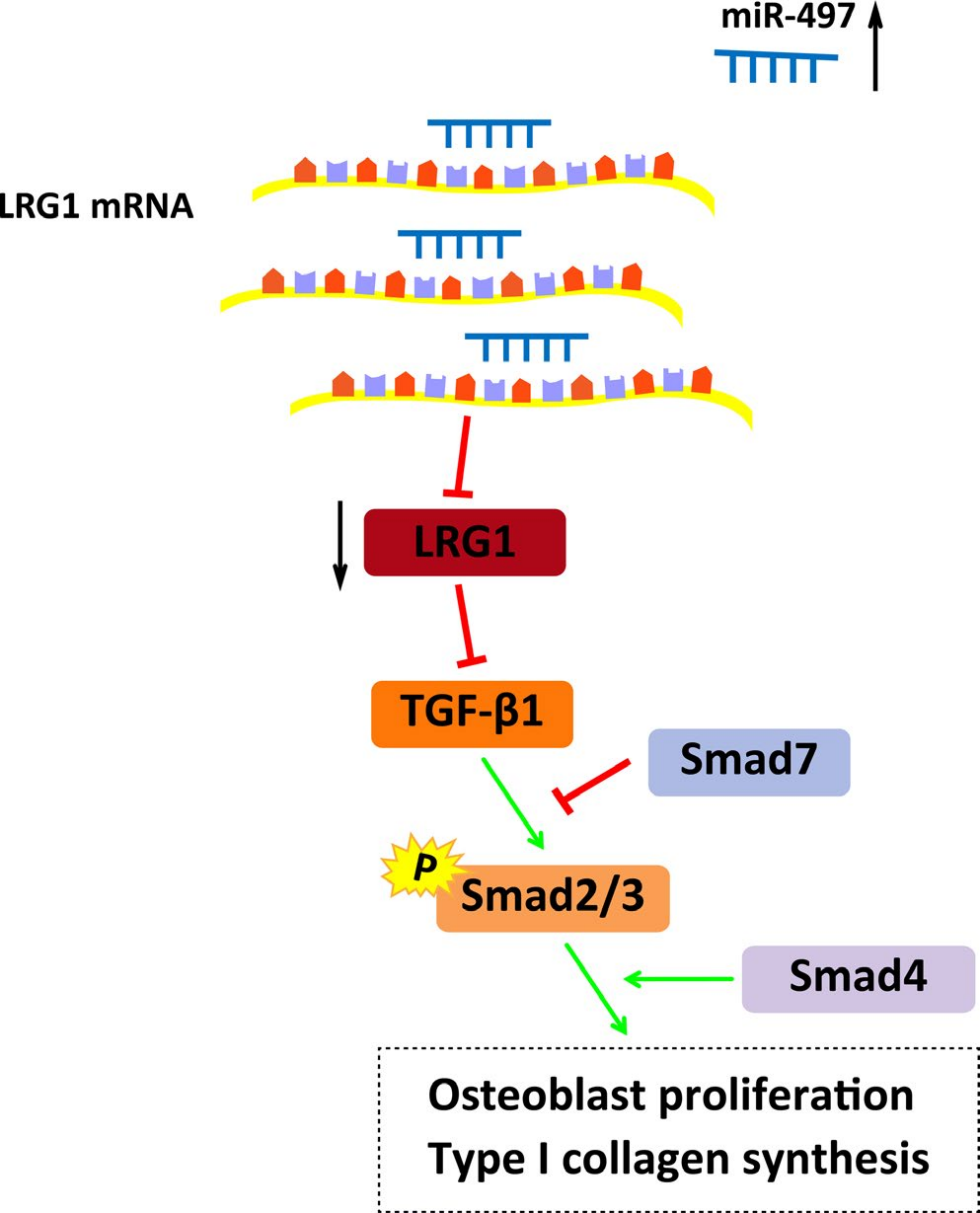
The mechanistic diagrams indicate that in osteoporosis, miR‐497 could promote osteoblast proliferation and collagen synthesis, and inhibit osteoblast apoptosis through inhibiting LRG1 and activating TGF‐β1/Smads signalling pathway. The miR‐497 mimics inhibited the expression of the LRG1 gene and activated TGF‐β1/Smads signalling pathway, including elevated expression of TGF‐β1, Smad3, Smad4 and p‐Smad2/3, and decreased expression of Smad7, thereby promoting osteoblast proliferation and collagen synthesis, and suppressing osteoblast apoptosis

In conclusion, our study reveals that miR‐497 is down‐regulated and LRG1 is upregulated in osteoporosis, and the increased miR‐497 or decreased LRG1 acts to alleviate the progression of osteoporosis via activating TGF‐β1/Smads pathway (Figure 8). Therefore, more potential therapeutic strategies pivoting on miR‐497 and LRG1 are emerged. Further investigations of the mechanism of miR‐497 and LRG1 should be explored in detail and performed with a larger cohort, so as to support a promising clinical application for patients with osteoporosis.

## CONFLICT OF INTEREST

The authors declare that they have no conflicts of interest.

## AUTHORS’ CONTRIBUTION

ZhengTao Gu: Data curation (equal); Formal analysis (equal); Writing‐original draft (equal). DengHui Xie: Data curation (equal). CaiQiang Huang: Formal analysis (equal). Rui Ding: Data curation (equal). RongKai Zhang: Data curation (equal). QingChu Li: Data curation (equal); Formal analysis (equal). ChuangXin Lin: Formal analysis (equal). YiYan Qiu: Conceptualization (equal).

## ETHICAL STATEMENT

This study was approved and supervised by the animal ethics committee of the Third Affiliated Hospital, Southern Medical University, Academy of Orthopedics of Guangdong Province. The treatment of animals in all experiments conforms to the ethical standards of experimental animals.

## CONSENT FOR PUBLICATION

Not applicable.

## Data Availability

Not applicable.
